# The long noncoding RNA *HOXA11 antisense* induces tumor progression and stemness maintenance in cervical cancer

**DOI:** 10.18632/oncotarget.12863

**Published:** 2016-10-25

**Authors:** Hee Jung Kim, Kyung Jin Eoh, Lee Kyung Kim, Eun Ji Nam, Sun Och Yoon, Kun-Hong Kim, Jae Kwan Lee, Sang Wun Kim, Young Tae Kim

**Affiliations:** ^1^ Institute of Women's Life Medical Science, Department of Obstetrics and Gynecology, Yonsei University College of Medicine, Seoul, Korea; ^2^ Department of Obstetrics and Gynecology, Yonsei University Graduate School, Seoul, Korea; ^3^ Department of Pathology, Gangnam Severance Cancer Hospital, Yonsei University College of Medicine, Seoul, Korea; ^4^ Department of Biochemistry and Molecular Biology, Yonsei University College of Medicine, Seoul, Korea; ^5^ Department of Obstetrics and Gynecology, Korea University Guro Hospital, Korea University College of Medicine, Seoul, Korea

**Keywords:** HOXA11 antisense, long noncoding RNA, invasion, prognosis, cervical cancer

## Abstract

Recent research has focused on the impact of long noncoding RNA (lncRNA) in cervical carcinogenesis. However, whether *HOXA11 antisense* (*HOXA11-AS*) is involved in cervical cancer remains to be elucidated. In the present study, we examined *HOXA11-AS* expression levels in cervical cancer patients and determined the relationships between *HOXA11-AS* expression and clinicopathological factors. We also investigated the bio-functional consequences of *HOXA11-AS* overexpression both *in vitro* and *in vivo*. *HOXA11-AS* expression was significantly greater in tissues from patients with cervical cancer than in control patients (*P<0.001*). Multivariate analysis showed that high *HOXA11-AS* was an independent prognosticator of overall survival (Hazard ratio=2.450, *P=0.032*). *HOXA11-AS* overexpression enhanced cell proliferation, migration, and tumor invasion *in vitro*, whereas *HOXA11-AS* knockdown inhibited these biologic aggressive features. These adverse changes were accompanied by characteristics of epithelial-mesenchymal transition (EMT). *In vivo* xenograft experiments using the siHOXA11-AS-transfected HeLa cells revealed that *HOXA11-AS* strongly induced tumor growth. Furthermore, we found that *HOXA11-AS* knockdown decreased cancer stemness and triggered the EMT program. In conclusion, *HOXA11-AS* overexpression correlated with poor survival in patients with cervical cancer. Thus, *HOXA11-AS* may be a pivotal target for exploring novel cervical cancer therapeutics.

## INTRODUCTION

Cervical cancer is the third most common cancer and the fourth leading cause of malignancy related mortality in women worldwide [1]. Although widespread implementation of screening programs in recent years has decreased the incidence and mortality of this cancer, it continues to be a major public health problem, specifically in advanced cases [2]. Major research efforts have focused on identifying tumor-specific markers predicting the biological behavior of cervical cancers, because cell motility and invasion are crucial in the progression of cancer [3]. An increased understanding of the molecular mechanisms underlying cervical carcinogenesis and progression is required to identify reliable prognosticators of tumor aggressiveness.

Noncoding RNAs (ncRNAs) may be key factors in gene regulation, influencing normal and cancer cell phenotypes [4, 5]. Over 3000 human long intervening coding RNAs (lincRNAs), and most long ncRNAs, are associated with DNA-binding proteins such as chromatin-modifying complexes [6] that epigenetically regulate the expression of multiple genes [7]. Transcription of long noncoding RNAs (lncRNAs) modulates gene activity in response to external oncogenic stimuli and DNA damage [8].

Several cancers highly express the homeobox A11 antisense lncRNA (*HOXA11-AS*), which is near the homeobox A11 (*HOXA11*) gene, further supporting the model that this lncRNA influences cervical cancer progression [9]. Human *HOX* gene clusters feature prevalent intergenic transcription between coding genes [10]. Noncoding RNAs seem to dominate homeobox gene cluster intergenic transcripts, which include short microRNAs (miRNA) and lncRNAs that are antisense to their canonical *HOX* neighbors. In humans and mice, *HOX* transcription factors stimulate embryonic development [11]. Homeobox A11 antisense lncRNA transcripts occur in the adult human endometrium. The abundance of these transcripts varies throughout the menstrual cycle; peak antisense RNA levels occur in the midproliferative phase, varying inversely with mRNA expression levels. In primary stromal cell culture, progesterone down-regulates *HOXA11-AS* transcription. This *HOXA11-AS* downregulation is followed by *HOXA11* mRNA upregulation, indicating a possible role for the antisense transcript in regulating mRNA expression [12]. The mechanism by which *HOXA11-AS* represses *HOXA11* mRNA is transcriptional interference rather than sense/antisense interaction; *HOXA11-AS* represses *HOXA11* by competing for transcription of a common gene. Homeobox A11 DNA methylation prognosticates ovarian cancer [13]. Homeobox A11 antisense lncRNA suppresses the expression of the *HOXA11* gene. Although *HOXA11* DNA methylation was observed to correlate in the progression of ovarian cancer, little is known about the molecular mechanisms underlying cervical cancer.

Cancer stem cells (CSCs) are responsible for tumor-initiating capacity, invasion, metastasis, relapse, and chemotherapy resistance [14]. The presence of a small population of CSCs in cervical cancer has major implications for cancer therapy and the complete eradication of refractory tumors. According to the CSC theory, these cells exhibit high levels of resistance to multi-drug treatment, as they possess an increased capacity for proliferation and DNA repair, and a downregulated epithelial-mesenchymal transition (EMT) program [15, 16]. However, the complex biology of cervical CSCs and the underlying pathogenic mechanisms remain unknown. Recent studies focus on molecular mechanisms underlying cervical CSC progression and new therapies against cervical CSCs [17–19].

The present study investigated the expression and molecular function of *HOXA11-AS* in cervical cancer cell lines and cancer tissues. We also examined the role of *HOXA11-AS* in tumor progression and CSCs. The findings of this study will be useful in elucidating the role of *HOXA11-AS* in the metastatic progression of cervical cancer.

## RESULTS

### Elevated expression of *HOXA11-AS* correlates with poor cervical cancer prognosis

**Figure 1:**
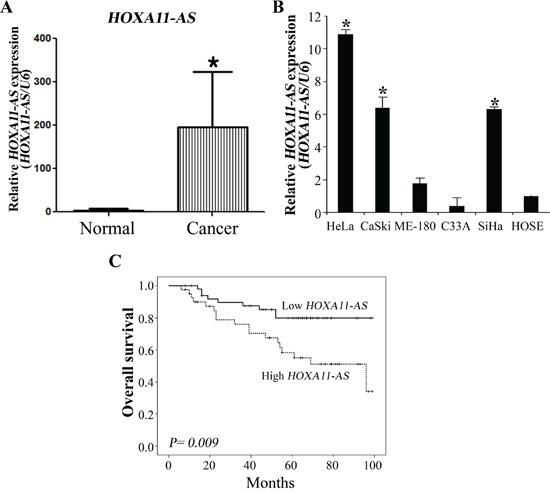
Relative *HOXA11-AS* expression and its clinical significance **A.** Elevated expression of *HOXA11-AS* in human cervical cancer tissues. Homeobox A11 antisense lncRNA expression was significantly higher in cervical cancer tissues (n=92) than in noncancerous tissues (n=30). Relative *HOXA11-AS* expression was determined using qRT-PCR with *U6* as an internal control. Data are expressed as means ± SD. *P<0.05 vs. non-tumor control. **B.** Expression of *HOXA11-AS* in cervical cancer cells. Homeobox A11 antisense lncRNA expression was evaluated using qRT-PCR with *U6* as an internal control. **C.** The median OS durations were 43 and 46.5 months in the high and low *HOXA11-AS* expression groups, respectively. The log-rank test indicated that the low *HOXA11-AS* expression group had a significantly longer OS than the high group (P=0.009). Five-year survival rates were 54.5% and 75.7% in the high and low *HOXA11-AS* expression groups, respectively.

Real time RT-PCR was performed to evaluate the expression of *HOXA11-AS* lncRNA in cervical cancer tissues (n=92) and corresponding normal tissues (n=30). Homeobox A11 antisense lncRNA expression in cervical cancer tissues was more than 227.5-fold that of noncancerous tissues (Figure 1A), suggesting that the expression of *HOXA11-AS* is upregulated in cervical cancer. We also performed real time RT-PCR assays on *HOXA11-AS* expression levels in six different cell lines, one of which was derived from human normal ovarian cells (HOSE), and five of which were derived from human cervical cancers. We found that *HOXA11-AS* expression levels were higher in epitheloid cervical carcinoma (HeLa), epidermoid cervical carcinoma established from a metastasis in the small bowel mesentery (CaSki), and squamous cervical carcinoma (SiHa) cells than in epidermoid cervical carcinoma (ME-180) and HPV negative cervical carcinoma (C33A) cells (Figure 1B).

We examined the relationship between *HOXA11-AS* expression and clinical outcomes (Table 1). Patients with high *HOXA11-AS* expression presented more lymphovascular invasion, lymph node metastasis, and recurrence relative to patients with low *HOXA11-AS* expression, but this relationship was not statistically significant.

**Table 1:** Clinicopathological features and ***HOXA11-AS*** expression in cervical cancer patients

Variables	Total (n=92)	High *HOXA11-AS* (n=41)	Low *HOXA11-AS* (n=51)	*p value*
Age (years)	51.5 ± 12.7	51.9 ± 12.6	51.2 ± 10.3	0.734
Stage				
I	36 (39.1%)	15 (36.6%)	21 (41.2%)	0.23
II	46 (50.0%)	19 (46.3%)	27 (52.9%)	
III	10 (10.9%)	7 (17.1%)	3 (5.9%)	
Histology				
Squamous cell	63 (68.5%)	28 (68.3%)	35 (68.6%)	0.098
Adenomatous	21 (22.8%)	10 (24.4%)	11 (21.6%)	
Mixed	2 (2.2%)	2 (4.9%)	0	
Small cell	5 (5.4%)	0	5 (9.8%)	
Unknown	1 (1.1%)	1 (2.4%)	0	
Lymphovascular invasion	46 (50.0%)	23 (56.1%)	23 (45.1%)	0.052
Lymph node metastasis	21 (22.8%)	12 (29.3%)	9 (17.6%)	0.142
Recurrence	24 (26.1%)	13 (31.7%)	11 (21.6%)	0.194

The median overall survival (OS) durations were 43 and 46.5 months in the high and low *HOXA11-AS* expression groups, respectively. The log-rank test indicated a significantly longer OS for the low *HOXA11-AS* expression group (P=0.009) (Figure 1C). Five-year survival rates were 58.3% and 79.9% in high and low *HOXA11-AS* expression groups, respectively. A Cox multivariate proportional hazards analysis showed that stage (hazard ratio [HR]=3.546, P=0.02), nodal metastasis (HR=2.724, p=0.023), and *HOXA11-AS* (HR=2.450, P=0.032) were independent prognosticators of overall survival (Table 2).

**Table 2:** Univariate and multivariate analyses for various determinants in patients with cervical cancer

	No. of patients	Univariate analysis		Multivariate analysis	
		Hazard ratio (95% CI)	p-value	Hazard ratio (95% CI)	p-value
Age, years (continuous)	92	0.993 (0.960-1.027)	0.692		
Stage					
1	36	1 (Reference)		1 (Reference)	
2	46	0.743 (0.314-1.760)	0.5	0.650 (0.254-1.659)	0.367
3	10	3.492 (1.258-9.691)	0.016	3.546 (1.223-10.280)	0.02
Lymphovascular invasion					
No	43	1 (Reference)			
Yes	46	2.118 (0.094-4.774)	0.07		
Nodal metastasis					
No	71	1 (Reference)		1 (Reference)	
Yes	21	2.617 (1.210-5.662)	0.015	2.724 (1.145-6.483)	0.023
Recurrence					
No	68	1 (Reference)		1 (Reference)	
Yes	24	3.268 (1.521-7.020)	0.002	2.046 (0.862-4.857)	0.105
*HOXA11-AS* expression					
Low	51	1 (Reference)		1 (Reference)	
High	41	2.769 (1.242-6.174)	0.013	2.450 (1.079-5.561)	0.032

### Knockdown of *HOXA11-AS* decreases cell proliferation in cervical cancer cells

**Figure 2:**
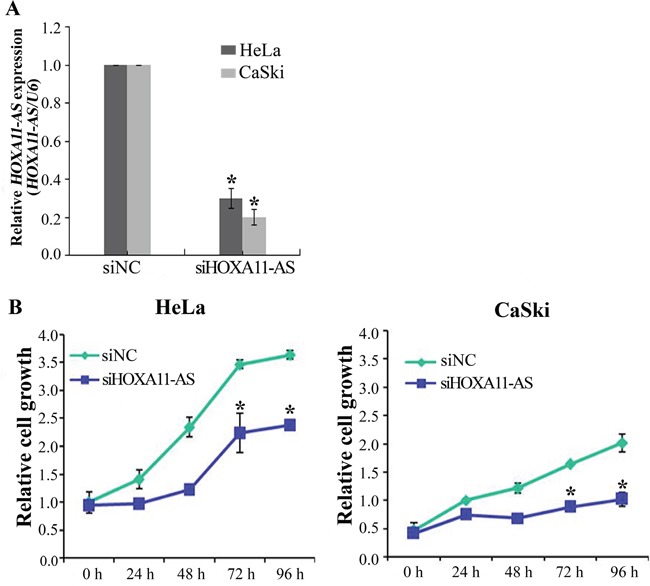
Knockdown of *HOXA11-AS* inhibits the proliferation of cervical cancer cells **A.** Cells were transfected with *HOXA11-AS*-specific siRNA and negative control siRNA (siNC), and knockdown efficiency was determined by qRT-PCR analysis. **B.** Knockdown of *HOXA11-AS* decreases cell proliferation in HeLa and CaSki cells. The proliferation of cervical cancer cells transfected with siHOXA11-AS and siNC was determined using the CCK-8 assay. Bars indicate means ± SD of three independent experiments. *P<0.05 vs. siNC.

To investigate the functional role of *HOXA11-AS* in cervical cancer, siRNA was used to downregulate *HOXA11-AS* expression. HeLa and CaSki cells were used for siRNA-mediated knockdown of *HOXA11-AS* expression. The knockdown efficiency of the *HOXA11-AS-*specific siRNAs (siHOXA11-AS) was evaluated, and siHOXA11-AS was found to have a higher silencing efficiency than the negative control siRNA (Figure 2A). We next examined the impact of *HOXA11-AS* knockdown on cell proliferation. The results of the CCK-8 assay showed that siRNA-mediated knockdown of *HOXA11-AS* in HeLa and CaSki cells decreased cell proliferation (Figure 2B), suggesting that *HOXA11-AS* is involved in the proliferation of cervical cancer.

### Lentiviral-mediated overexpression or knockdown of *HOXA11-AS* reveals that high *HOXA11-AS* expression promotes cervical cancer cell migration and invasion

**Figure 3:**
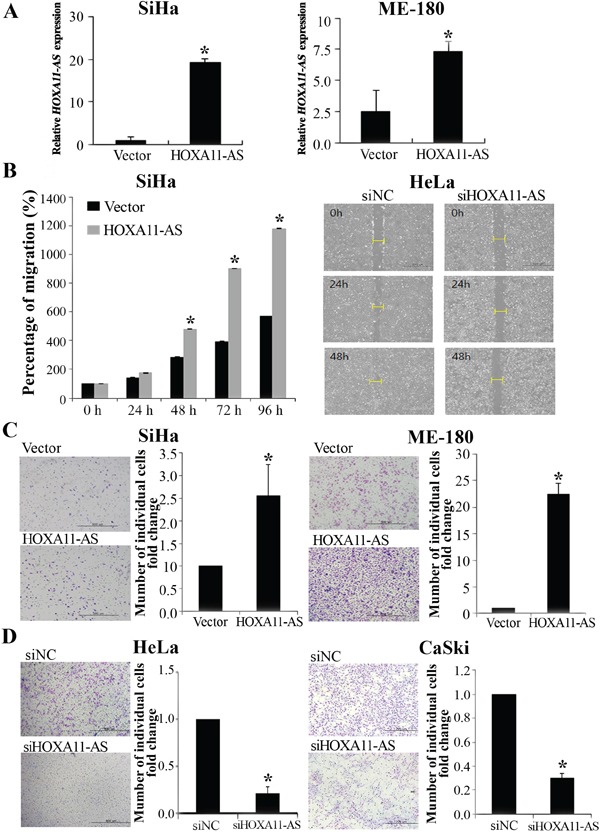
Homeobox A11 antisense lncRNA promotes cell migration and invasion **A.** Overexpression of *HOXA11-AS* in SiHa and ME-180 cells, analyzed using qRT-PCR. **B.** Wound healing assay was used to determine migration in *HOXA11-AS* overexpressed SiHa cells and siHOXA11-AS-transfected HeLa cells (×200). **C.** Using the Matrigel invasion chamber, overexpression of HOXA11-AS in SiHa and ME-180 cells increased the invasive capacity after 48 h. **D.** Matrigel invasion assay was used to determine invasion after 48 h in siHOXA11-AS-transfected HeLa and CaSki cells. Each assay was performed in triplicate. Data are mean ± SD. *P<0.05 vs. control.

To determine whether *HOXA11-AS* increases migration and invasion in cervical cancer cells, we performed wound healing and Matrigel invasion assays. We established stable *HOXA11-AS*-overexpressed SiHa and ME-180 cells by antibiotic selection of a pool of lentivirus-infected cells (Figure 3A). Overexpression of *HOXA11-AS* resulted in increased migration of SiHa cells relative to empty vector-expressing controls (Figure 3B). In contrast, siRNA-mediated knockdown of *HOXA11-AS* inhibited cell migration in HeLa cells (Figure 3B). To investigate whether *HOXA11-AS* could also enhance invasion in cervical cancer cells, we performed a Matrigel invasion assay. Homeobox A11 antisense lncRNA overexpression in SiHa and ME-180 cells resulted in increased invasion relative to empty vector-expression cells (Figure 3C). In contrast, *HOXA11-AS* knockdown in HeLa and CaSki cells led to a decrease in cell invasion (Figure 3D).

### Knockdown of *HOXA11-AS* inhibits MMP-9, MMP-2, and VEGF expression in cervical cancer cells

**Figure 4:**
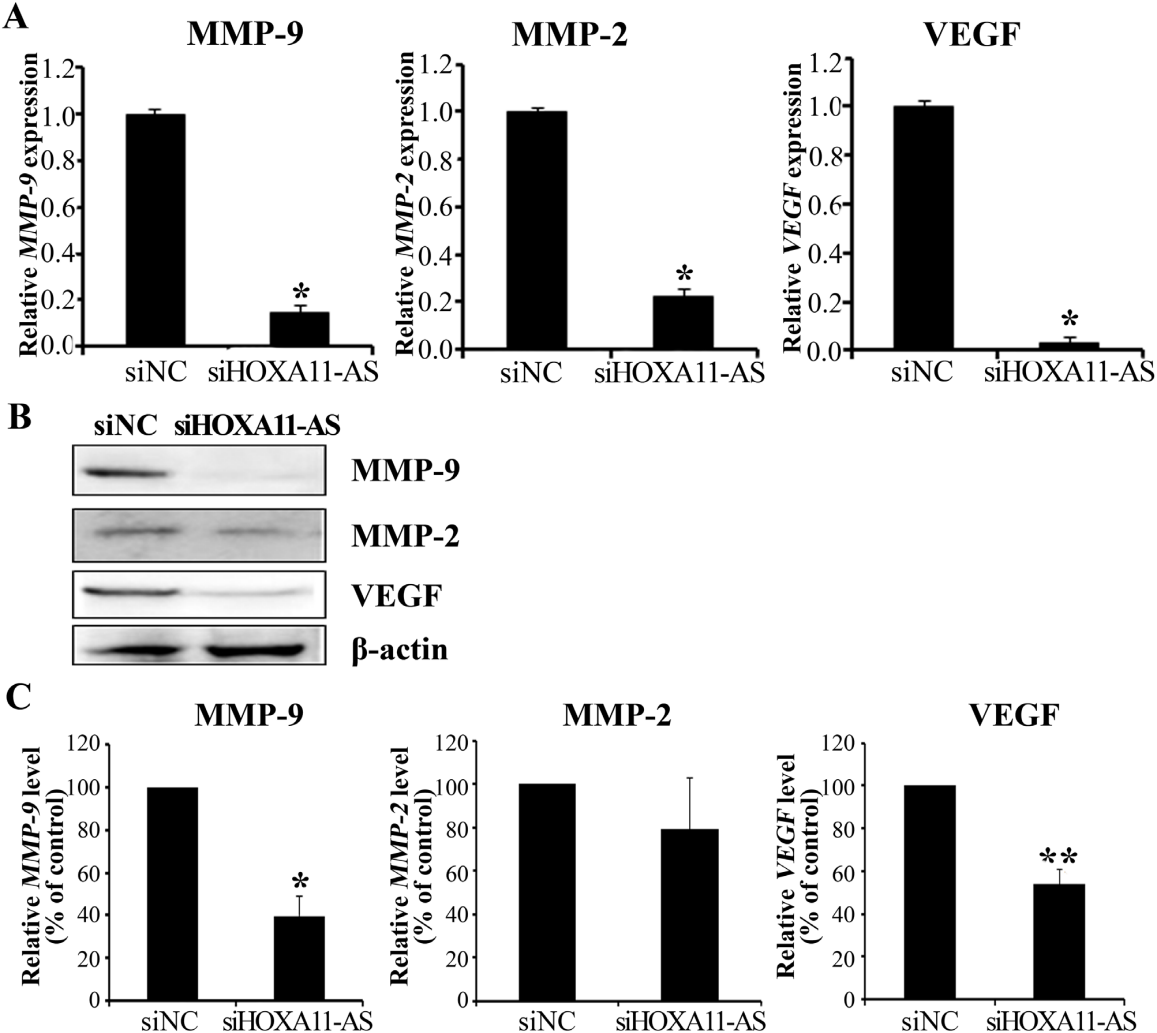
Knockdown of *HOXA11-AS* decreases MMP-9, MMP-2, and VEGF expression in cervical cancer cells **A.** MMP-9, MMP-2, and VEGF expression were analyzed by qRT-PCR. **B.** Protein lysates were obtained from siHOXA11-AS and siNC-transfected HeLa cells 48 h post-transfection. MMP-9, MMP-2, and VEGF expression were analyzed by western blotting. **C.** Band intensities were quantified and normalized to that of β-actin. Each assay was performed in triplicate. Data are mean ± SD. *P<0.05 vs. siNC. **P<0.05 vs. siNC.

To explore molecular mechanisms underlying *HOXA11-AS* promotion of cell migration and invasion, we characterized the expression of MMP-9, MMP-2, and VEGF. siHOXA11-AS decreased MMP-9, MMP-2, and VEGF expression levels in HeLa cells (Figure 4A). siRNA-mediated knockdown of *HOXA11-AS* inhibited MMP-9, MMP-2, and VEGF protein expression (Figure 4B). Our findings suggest that *HOXA11-AS* promotes cervical cancer cell migration and invasion *via* upregulation of MMP-9, MMP-2, and VEGF.

### *HOXA11-AS* knockdown reversed EMT-related genes in cervical cancer cells

**Figure 5:**
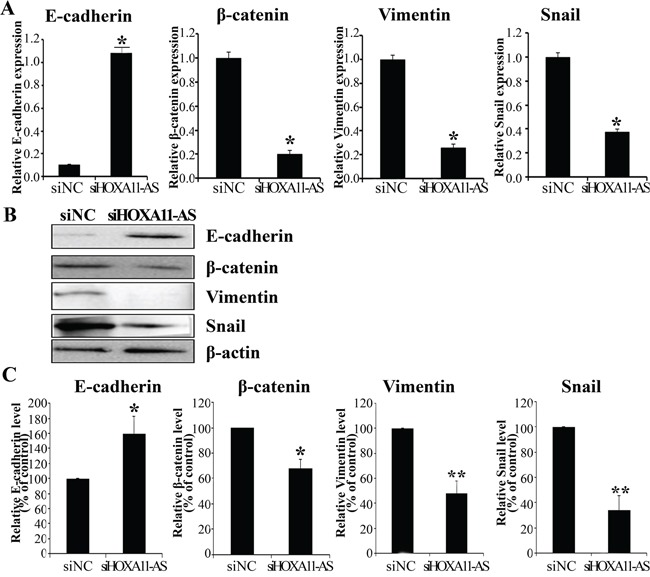
Expression of HOXA11-AS knockdown on the EMT-related genes in HeLa cells HeLa cells were transfected with *HOXA11-AS*-specific siRNA and siNC for 48 h. E-cadherin, β-catenin, Vimentin, and Snail expression were analyzed by qRT-PCR **A.** and western blotting **B.**, **C.** Band intensities were quantified and normalized to that of β-actin. Each assay was performed in triplicate. Data are mean ± SD. *P<0.05 vs. siNC. **P<0.05 vs. siNC.

Because the EMT is important in cell migration and invasion, we tested whether *HOXA11-AS* is required for EMT. We performed siRNA-mediated knockdown of *HOXA11-AS* in HeLa cells, and monitored EMT using real-time RT-PCR and western blot assays. Knockdown of *HOXA11-AS* resulted in an increase in E-cadherin expression and decreases in β-catenin and vimentin expression (Figures 5A–5B). In addition, the EMT-mediating transcription factor Snail was downregulated in siHOXA11-AS-transfected cells, relative to its level in negative control siRNA (siNC) transfected cells (Figures 5A–5B). These data suggest that dysregulation of EMT-related genes partially explains the involvement of *HOXA11-AS* in cervical cancer cell migration and invasion.

### *HOXA11-AS* promotes sphere formation and expression of stemness markers

**Figure 6:**
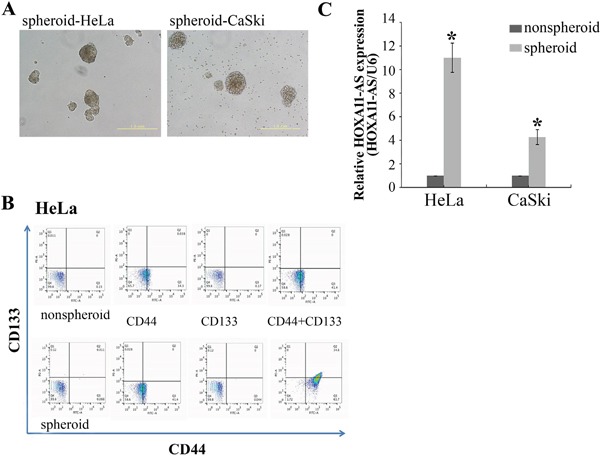
Sphere formation increased the CD133^+^/CD44^+^ cancer stem cell subpopulation and *HOXA11-AS* expression in cervical cancer cell lines **A.** HeLa and CaSki cells were incubated for 5 days, and stained with anti-CD44 and anti-CD-133. Representative dot-plots are shown in **B.**, **C.** Enriched populations of spheroid and non-spheroid HeLa and CaSki cells were subjected to qPCR analysis of *HOXA11-AS*. Each assay was performed in triplicate. Data are mean ± SD.

The EMT program confers stem cell-like properties to normal and tumor cells [16, 20, 21]. To assess whether *HOXA11-AS* promotes CSC generation, we treated the CaSki and HeLa cervical cancer cells for 5 days, and counted the number of cells expressing the CSC markers CD133^+^/CD44^+^ [22, 23]. Spheroid HeLa cells promoted an increase in the number of CD133^+^/CD44^+^ (Figure 6A and 6B). We next evaluated *HOXA11-AS* expression in non-spheroid and spheroid cells. Gene expression analyses in CaSki and HeLa cells showed that spheroid cells express *HOXA11-AS* at levels tenfold and fourfold that of control cells, respectively (Figure 6C).

**Figure 7:**
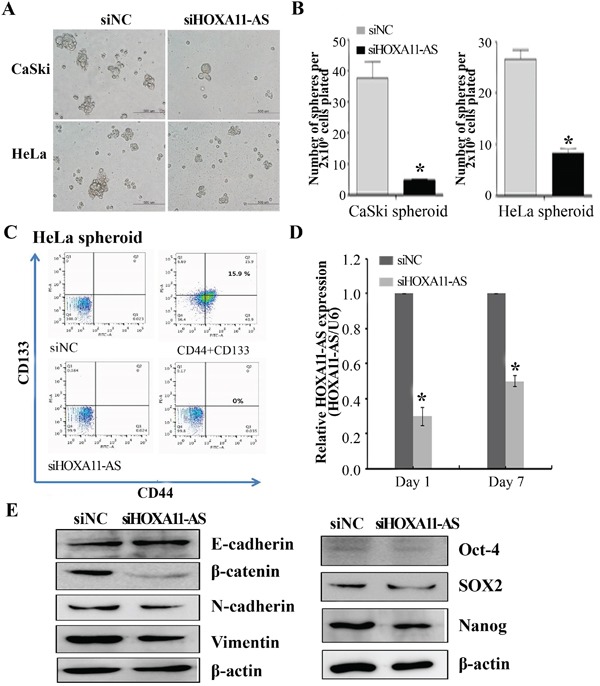
Homeobox A11 antisense lncRNA knockdown inhibited sphere formation Representative images **A.** and quantification **B.** of spheres formed from CaSki and HeLa cells, previously transfected with siHOXA11-AS or siNC. Spheres were counted by visual inspection in light microscopy. HeLa cells were incubated for 7 days, and stained with anti-CD44 and anti-CD-133. Representative dot-plots are shown in **C.**, **D.** qPCR was performed to evaluate the expression of *HOXA11-AS* on days 1 and 7 of the sphere assays. Data are reported as means ± SD for three independent experiments. *P<0.05 vs. siNC. **E.** EMT-related gene, Oct-4, SOX2, and Nanog expression were analyzed by western blotting.

Next, to determine whether *HOXA11-AS* promotes self-renewal of cervical cancer cells, an important feature of CSCs, we assayed sphere formation in CaSki and HeLa cells 2 days after *HOXA11-AS* knockdown. After 7 days of incubation in anchorage-independent conditions, the control cells presented an elevated number of large colonies, while the *HOXA11-AS*-silenced cells formed only a few colonies of small size (Figure 7A). Counting of colonies revealed a reduction in sphere formation (Figure 7B). We counted the number of cells expressing the CD133^+^/CD44^+^ CSC markers. As expected, siHOXA11-AS-spheroid HeLa cells exhibited a decrease in the number of CD133^+^/CD44^+^ compared with the siNC-spheroid HeLa cells (Figure 7C). In order to verify the effectiveness of siHOXA11-AS during the sphere formation assays, we measured its expression 7 days after siRNA treatment. Although we detected a slight recovery in *HOXA11-AS* expression, a significant knockdown was still observed (Figure 7D). To further characterize siHOXA11-AS-decreased spheres and determine the molecular mechanisms underlying these observations, we evaluated the expression of several stemness genes in siHOXA11-AS-spheroid HeLa cells using western blotting (Figure 7E). We found that the expression of SOX2, Oct-4, and Nanog were significantly downregulated in siHOXA11-AS-spheroid HeLa cells, relative to the corresponding expression levels in control cells.

Since *HOXA11-AS* is necessary to promote the EMT program (Figure 5), we evaluated the expression of EMT-related genes using western blot analysis in siHOXA11-AS-spheroid HeLa cells. β-Catenin, N-cadherin, and vimentin expression was downregulated in siHOXA11-AS-spheroid HeLa cells, relative to the corresponding levels in control cells (Figure 7E). These results suggest that *HOXA11-AS* contributes to activating the genetic program that promotes EMT and supports the CSC phenotype.

### Knockdown of *HOXA11-AS* decreases xenograft tumor growth in mice

**Figure 8:**
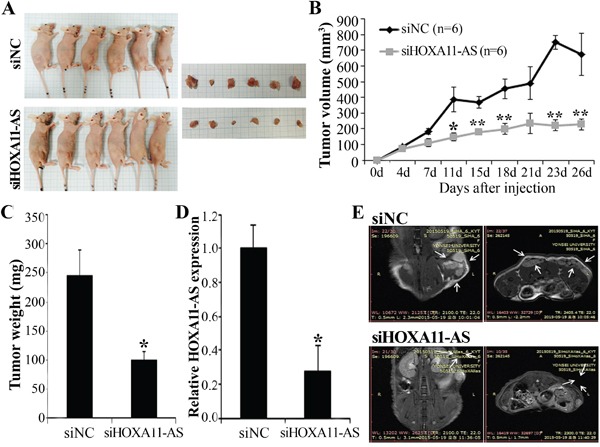
Effect of *HOXA11-AS* on tumor growth *in vivo* **A.** Images represent tumor growth in the nude mice 26 days after they were injected with 5 × 10^6^ siNC or siHOXA11-AS-transfected HeLa cells (n=6 mice/group). Gross images of tumor masses from representative mice from each group (right panel). **B.** Tumor volume was calculated every 3~4 days. Data are mean ± SE (n=6). *P<0.05 and ** P<0.001 vs. siNC. **C.** Tumor weight. Data are mean ± SE. **D.** qRT-PCR analysis of *HOXA11-AS* expression in tissues of resected tumors. **E.** Representative MRI imaging of mice four weeks after injection.

To assess whether *HOXA11-AS* knockdown can decrease tumor growth *in vivo*, we inoculated HeLa cells as xenografts into nude mice (Figure 8A). Tumor volume and weight were measured. Mice injected with siHOXA11-AS-transfected cells presented significantly decreased tumor growth and weights compared with those injected with control cells (Figures 8B–8C). Homeobox A11 antisense lncRNA expression in tumor tissue was downregulated in siHOXA11-AS-transfected cells compared with control cells (Figure 8D). Tumor weight correlated with tumor volume, as determined by calipers (P<0.001; r^2^=0.935). We further evaluated tumor size using MRI (Figure 8E). Tumor size was strongly inhibited by *HOXA11-AS* knockdown. These findings suggested that *HOXA11-AS* promoted tumor growth *in vivo* and further supported our hypothesis that *HOXA11-AS* is involved in the pathogenesis of cervical cancer cells.

## DISCUSSION

Long noncoding RNAs are protagonists in recent narratives of the complex mechanisms underlying malignant processes including tumorigenesis, drug-resistance, and metastasis [24–26]. The present study investigated the molecular function and clinical significance of *HOXA11-AS* expression in cervical cancer cell lines and mouse xenograft models. We found that enhanced *HOXA11-AS* expression was associated with poor overall survival. Knockdown of *HOXA11-AS* expression correlated with decreased cell growth, migration, and invasion in cervical cancer cells. The effects of *HOXA11-AS* on tumor progression may be mediated by genes involved in cell migration, invasion, and EMT-related genes; these genes include VEGF, MMP-9, MMP-2, E-cadherin, β-catenin, Vimentin, and Snail. In addition, downregulation of *HOXA11-AS* expression inhibits EMT-related genes and the colony forming capacity of cervical cancer cells. Our findings indicate that *HOXA11-AS* may serve as a biomarker and therapeutic target for cervical cancer.

Long noncoding RNAs are transcripts of at least 200 nucleotides without protein-coding capacity. Little is known about the regulatory roles of lncRNAs and their relevance to human disease; however, the functional role of small regulatory ncRNAs such as miRNAs in human cancers is well established. Many lncRNAs are capped, spliced, and polyadenylated, like their protein-coding counterparts [27]. Long noncoding RNAs exhibit tissue-specific expression patterns. The growing list of functionally characterized lncRNAs implies that these transcripts are critical to various physiological processes [28], and therefore, modified expression of lncRNAs may affect cancer development and progression [29].

The literature increasingly reports that Hox proteins correlate with cancer development [9, 30, 31]. Homeobox genes were first discovered in *Drosophila melanogaster*, and subsequently the structures of human *HOX* proteins were solved [37, 38]. Several cancers that overexpress *HOXA11* include epithelial ovarian cancers [13, 30], bladder cancer [32], cervical cancer [9] and glioma [36]. However, there are few reports of the biology and function of *HOXA11-AS* in cervical cancer cells.

We found that *HOXA11-AS* expression associates with disease progression in cervical cancer patients; this lncRNA increased the proliferation, migration, and invasion of cervical cancer cells *in vitro* and *in vivo*. Long noncoding RNAs are essential for the regulation of chromatin structure, gene expression, and translational control [33]. In particular, the level of *HOXA11* methylation efficaciously distinguishes high-grade squamous intraepithelial lesion cells from healthy cells; *HOXA11* is also important in the embryological development of the Müllerian duct in the uterine cervix [34]. Through *HOXA11*, *HOXA11-AS* may affect cervical cancer development.

In addition, we discovered that downregulation of *HOXA11-AS* expression decreases cervical cancer cell proliferation, migration, and invasion. Therefore, *HOXA11-AS* may exert oncogenic activity in cervical cancer, and promote aggressive and metastatic characteristics. MMP-9 degrades basement membrane collagen, and accordingly promotes tumor cell invasion and metastasis, decreasing survival in many types of cancer [35, 36]. Moreover, tumor angiogenesis is decisive in tumor growth, invasion, and metastasis. The angiogenic factor VEGF activates tumor angiogenesis, and accordingly is a major target of many cancer medications [37]. In our investigation, high expression of *HOXA11-AS* in cervical cancer cell induced cell migration and invasion through the upregulation of EMT-related genes, VEGF, and MMP-9. More importantly, downregulation of *HOXA11-AS* expression prevented EMT induction and CSCs arising. We found that *HOXA11-AS* increases sphere-forming capacity. Furthermore, *HOXA11-AS* knockdown decreased expression of stemness genes, SOX2, Oct-4, and Nanog in cervical cancer cells. In addition, knockdown of *HOXA11-AS* downregulated expression of EMT-related genes. We hypothesized that *HOXA11-AS* acts as a key regulator of different signaling mechanisms involved in EMT/stemness establishment. We provided the first evidence that *HOXA11-AS* promotes CSC self-renewal and EMT in cervical cancer cells, which may contribute to cervical cancer growth, invasion, and recurrence.

The recurrence rate after radical surgery for early-stage cervical cancer is 15–30%, and the recurrent patients have poor prognoses [38]. Reliable predictors of recurrence and progression are necessary to improve the prognoses of cervical cancer patients. Pelvic lymph node metastasis is one of the most important postoperative risk factors for recurrence or failure to survive. Thus, cervical cancer patients with metastasis in the pelvic lymph nodes require adjuvants such as postoperative radiotherapy [2, 36].

We show that high *HOXA11-AS* expression correlates with poor overall survival, as the advanced stage and nodal metastasis. Evaluation of *HOXA11-AS* expression in cervical cancer patients may predict the risk of progression or recurrence, thereby informing treatment decisions. Notwithstanding the prognostic significance of *HOXA11-AS* for tumor progression or recurrence, the present study's relatively small sample size signals for interpretive caution. Larger prospective studies are necessary to validate our findings.

## MATERIALS AND METHODS

### Patient specimens

Included in this study were 92 female patients who underwent surgery between 2007 and 2014 at Yonsei Severance Hospital, Yonsei University. Specimens from patients with newly diagnosed invasive stage IA to IVB cervical cancer (International Federation of Gynecology and Obstetrics, FIGO) who had not received prior treatment were included in this study. Thirty samples of normal cervix from patients undergoing simple hysterectomy because of uterine leiomyomata were obtained as controls. This study was conducted according to the principles of the Declaration of Helsinki, and was approved by the Ethics Committee of Yonsei Severance Hospital. Informed consent was obtained from all patients. All specimens were immediately frozen in liquid nitrogen and stored at –80°C until RNA extraction.

### Cell lines

We obtained SiHa, HeLa, CaSki, ME-180 and C33A human cervical cancer cell lines from the American Type Culture Collection (Rockville, MD). Squamous cervical carcinoma and HeLa cells were cultured in Dulbecco's Modified Eagle Medium; ME-180 and C33A cells were cultured in Eagle's Minimum Essential Medium (Gibco-BRL, Gaithersburg, MD, USA) ; CaSki cells were cultured in RPMI-1640 medium (Gibco-BRL). The human normal ovarian cancer cell line HOSE was cultured in MCDB 105 (Sigma Aldrich, Castle Hill, Australia) medium. Culture media were supplemented with 10% (v/v) fetal bovine serum and penicillin/streptomycin. All cell lines were maintained at 37°C in a humidified incubator with 5% CO_2_.

### Quantitative real-time PCR (qRT-PCR)

Total RNA was extracted using TRIzol® reagent (Invitrogen Corp., Carlsbad, CA, USA) according to the manufacturer's instructions. Two micrograms of total RNA were reverse transcribed into first-strand cDNA using a reverse transcription reagent kit (Invitrogen). The cDNA template was amplified by qRT-PCR, using the SYBR® Green Real-time PCR Kit (TOYOBO Co. Ltd, Osaka, Japan). qRT-PCR was performed on the ABI StepOnePlus Real-Time PCR System (Applied Biosystems, Foster City, CA, USA). All quantifications were performed with *U6* as the internal standard. Relative gene expression was analyzed using the 2–ΔΔ*C*T method, and the results were expressed as extent of change with respect to control values. qRT-PCR experiments were replicated at least three times. Primers used for PCR reactions are shown in Table S1.

### Small interfering RNA (siRNA) transfection

Homeobox A11 antisense lncRNA siRNA (siHOXA11-AS) and negative control siRNA (siNC) were purchased from Genolution (Genolution Pharmaceuticals Inc., Seoul, Korea). Cells (5×10^4^ cells/well) were seeded into 6-well plates and transfected with 10 nM siRNA in phosphate-buffered saline (PBS), using the G-Fectin Kit (Genolution Pharmaceuticals), according to the manufacturer's protocol. These siRNA-transfected cells were used in the *in vitro* assays 48 h post-transfection. The target sequences for *HOXA11-AS* siRNAs were as follows: siRNA, 5′-CGGAAUAUCGGAAUAAAGUUU-3′. The experiments were repeated at least three times.

### Plasmid constructs and the generation of stable cell lines

Full-length human HOXA11-AS transcript cDNA was amplified by PCR and inserted into the pLenti6/V5-D-TOPO vector, according to the ViraPower™ Lentiviral Expression Systems (Invitrogen) protocol. The plasmid was transfected into 293FT cells for packaging, and the resultant lentivirus was used to infect the desired cell lines. The selection of *HOXA11-AS* stably transfected cells was performed in medium containing blasticidin (Invitrogen).

### Cell proliferation assay

Cell proliferation was evaluated using the Cell Counting Kit-8 (CCK-8) assay (Dojindo Laboratories, Kumamoto, Japan). Cells (2 × 10^3^ cells/well) were seeded into 96-well flat-bottomed plates in 100 μL of complete medium. The cells were incubated overnight to allow for cell attachment and recovery, and were subsequently transfected with siNC or siHOXA11-AS for 24, 48, 72, or 96 h. An aliquot of 10 μL of CCK-8 solution was added to each well and incubated for 2 h. The absorbance was measured at 450 nm to calculate the number of viable cells in each well. Three independent experiments were performed in triplicate.

### Matrigel invasion assay

The Matrigel invasion assay was performed using the BD Biocoat Matrigel Invasion Chamber (pore size: 8 mm, 24-well; BD Biosciences, Bedford, MA, USA), according to the manufacturer's protocol. Briefly, 5 × 10^4^ cells were plated in the upper chamber on serum-free medium, and complete medium was added to the bottom chamber. The Matrigel invasion chamber was incubated for 48 h at 37°C under 5% CO_2_. Non-invading cells were removed from the upper chamber using cotton-tipped swabs. Cells that had invaded through the pores onto the lower side of the filter were stained (Diff Quik, Sysmes, Kobe, Japan), and these cells were counted using a hemocytometer. The assay was replicated at least three times.

### Wound healing migration assay

Cell migration was assessed by wound healing assay. In brief, 5 × 10^5^ cells were seeded into 6-well culture plates with serum-containing medium and allowed to grow to 90% confluency in complete medium. The serum-containing medium was removed, and cells were serum starved for 24 h. When the cell density reached ~ 100% confluence, an artificial homogenous wound was created by scratching the monolayer with a sterile 200-μL pipette tip. After scratching, the cells were washed with serum-free medium. Images of cells migrating into the wound were captured at 0, 24, and 48 h using a microscope. The assay was performed in triplicate.

### Self-renewal assay

CaSki and HeLa cells (1 cell/μL in SFM) were seeded at 100 μL/well in 96-well plates for seven days. The total number of spheres in each well was counted under a microscope. The cells were dissociated, stained with Trypan blue (Amresco Inc., Solon, OH), and counted under a microscope to determine the total cell number. All experiments were done in triplicate.

### Fluorescence-activated cell sorting analysis

Cells were washed with PBS and fixed with PBS supplemented with 3% paraformaldehyde (10 min, 4°C), and incubated with one of the following four antibody treatments: no treatment; anti-CD44-APC (clone-IM7, eBioscience); anti-CD133-PE (clone-AC133, Miltenyi Biotec., San Diego, CA, USA); or both anti-CD133-PE and anti-CD44-APC. Cell staining was in accordance with manufacturer's instructions. FACS analysis was carried out with a FACScanto apparatus (Becton Dickinson).

### Western blot analysis

Proteins were extracted with RIPA buffer (Thermo Fisher Scientific Inc. Waltham, MA USA). Protein concentrations were measured using the Pierce BCA Protein assay kit (Thermo Fisher Scientific). After boiling with 2× sample buffer, proteins were resolved on 10% SDS–polyacrylamide gels, and transferred electrophoretically to polyvinylidene difluoride membranes (Millipore, Billerica, MA, USA). Membranes were blocked with 5% non-fat dried milk in 1× Tris-buffered saline containing 0.1% Tween 20 (pH 7.6) at room temperature for 1 h, and were subsequently incubated with primary antibody at 4°C overnight under constant agitation. The primary antibodies included rabbit anti-human VEGF (1:500 dilution; Abcam, Cambridge, MA, USA), rabbit anti-human MMP-2 (1:500 dilution; Abcam), rabbit anti-human MMP-9 (1:1000 dilution; Cell Signaling, Beverly, MA, USA), rabbit anti-human E-cadherin (1:1000 dilution; Cell Signaling), rabbit anti-human β-catenin (1:1000 dilution; Cell Signaling), mouse anti-human Vimentin (1:1000 dilution; Sigma, St. Louis, MO, USA), mouse anti-human Snail (1:1000 dilution; Cell Signaling), rabbit anti-human SOX-2 (1:1000 dilution; Cell Signaling), rabbit anti-human Nanog (1:1000 dilution; Cell Signaling), rabbit anti-human Oct-4 (1:1000 dilution; Cell Signaling), and mouse anti-human β-actin antibody (1:5000 dilution; Sigma). Proteins were visualized using an enhanced chemiluminescence system (ECL™; Amersham, Little Chalfont, UK), and band intensities were quantified using the Luminescent Image Analyzer (LAS-4000 mini, Fujifilm, Uppsala, Sweden).

### Xenografts in mice

BALB/c mice (n=12, 5–6 weeks of age, Orient Bio, Seongnam, Korea) were kept in aseptic, constant temperature and humidity, conditions (Yonsei Medical University protocol). Each mouse received a 150-μL subcutaneous dorsal scapula injection of HeLa cell suspension. The size of the tumor was measured twice weekly with calipers, and the tumor volume was determined using the simplified formula for a rotational ellipsoid (length × width^2^ × 0.5). Each tumor was harvested at 30 days post-treatment.

### Magnetic resonance (MR) imaging in mice

A Bruker Biospec 94/24 USR (9.4T) small animal scanner (35-mm diameter birdcage coil, Bruker BioSpin MRI, Ettlingen, Germany) was used to obtain the MR images. During the MR experiments, mice were immobilized by placement in a custom-built cradle. T_2-_weighted images were obtained at the beginning of each imaging session for accurate positioning of the animal inside the magnet bore. The T_2_-weighted images were acquired using the rapid acquisition setting. An O_2_/N_2_O mixture (1:1) with 1.5% isoflurane, at a 0.7 L/min flow rate, was used for anesthesia. An air pillow was used to monitor respiration. Circulating warm water was used to maintain mouse body temperature within acceptable limits.

### Comparison of clinical outcomes and survival

Low *HOXA11-AS* expression was defined as expression less than 10-fold. Ten-fold or more than 10-fold expression of *HOXA11-AS* was considered high HOXA11-AS. Survival rate and clinical outcomes were analyzed and compared between the two groups. Overall survival was defined as the period in months between the date of diagnosis and the date of death or last contact.

### Statistical analysis

IBM SPSS version 20 for Windows (SPSS Inc., Chicago, IL, USA) was used for the statistical analysis. The Kolmogorov–Smirnov test was used to verify standard normal distributional assumptions. Statistical significance was determined using Fisher's exact test, Pearson's chi square test, and Student's *t* test. Univariate and multivariate analysis using the Cox proportional hazards model was performed to assess the influence of various prognostic factors on survival. Survival outcomes were determined through a Kaplan–Meier survival analysis. Mean differences were considered significant when P<0.05. Results are shown as mean ± SD.

## SUPPLEMENTARY TABLE


