# Supplementary material for: The long noncoding RNA HOXA11 antisense induces tumor progression and stemness maintenance in cervical cancer
Source: Oncotarget. 2016 Oct 25;7(50):83001–16. doi: 10.18632/oncotarget.12863 (PMC5347748; doi:10.18632/oncotarget.12863)
Supplement: Supplementary file 1 [file oncotarget-07-83001-s001.pdf]

The long noncoding RNA *HOXA11 antisense* induces tumor progression and stemness maintenance in cervical cancer

SUPPLEMENTARY TABLE

Supplementary Table S1: Primer sequences used in this study

Gene	Primer sequence		Product Size (bp)
	Forward (5′-3′)	Reverse (5′-3′)	
HOXA11-AS	GAGTTTGAAGCCGTGGATGT	AGATGAGGGGAGAGGTGGAT	665
MMP-9	CGCTACCACCTCGAACTTTG	GCCATTCACGTCGTCCTTAT	196
MMP-2	GGATGATGCCTTTGCTCG	ATAGGATGTGCCCTGGAA	487
VEGF	TTGCTGCTCTACCTCCAC	AAATGCTTTCTCCGCTCT	419
E-cadherin	ATTCTGATTCTGCTGCTCTTG	AGTAGTCATAGTCCTGGTCCT	421
β-catenin	TGCAGTTCGCCTTCACTATG	ACTAGTCGTGGAATGGCACC	162
N-cadherin	CCCAAGACAAAGAGACCCAG	GCCACTGTGCTTACTGAATTG	140
Vimentin	TGGATTCACTCCCTCTGGTT	GGTCATCGTGATGCTGAGAA	111
Snail	GAGGCGGTGGCAGACTAG	GACACATCGGTCAGACCAG	178
Sox-2	AAGAGAACACCAATCCCATCCA	AGTCCCCCAAAAAGAAGTCCA	95
Nanog	GATTTGTGGGCCTGAAGAAA	TTGGGACTGGTGGAAGAATC	138
Oct-4	GCAAAGCAGAAACCCTCGTG	GAACCACACTCGGACCACAT	173
